# The Influence of Mucus Microstructure and Rheology in *Helicobacter pylori* Infection

**DOI:** 10.3389/fimmu.2013.00310

**Published:** 2013-10-10

**Authors:** Rama Bansil, Jonathan P. Celli, Joseph M. Hardcastle, Bradley S. Turner

**Affiliations:** ^1^Department of Physics, Boston University, Boston, MA, USA; ^2^Department of Physics, University of Massachusetts Boston, Boston, MA, USA; ^3^Division of Gastroenterology, Beth Israel Deaconess Medical Center, Harvard Medical School, Boston, MA, USA

**Keywords:** *H. pylori*, gastric mucosa, mucins, rheology, motility, atomic force microscopy, particle tracking microrheology, bacterial infections

## Abstract

The bacterium *Helicobacter pylori* (*H. pylori*), has evolved to survive in the highly acidic environment of the stomach and colonize on the epithelial surface of the gastric mucosa. Its pathogenic effects are well known to cause gastritis, peptic ulcers, and gastric cancer. In order to infect the stomach and establish colonies on the mucus epithelial surface, the bacterium has to move across the gel-like gastric mucus lining of the stomach under acidic conditions. In this review we address the question of how the bacterium gets past the protective mucus barrier from a biophysical perspective. We begin by reviewing the molecular structure of gastric mucin and discuss the current state of understanding concerning mucin polymerization and low pH induced gelation. We then focus on the viscoelasticity of mucin in view of its relevance to the transport of particles and bacteria across mucus, the key first step in *H. pylori* infection*.* The second part of the review focuses on the motility of *H. pylori* in mucin solutions and gels, and how infection with *H. pylori* in turn impacts the viscoelastic properties of mucin. We present recent microscopic results tracking the motion of *H. pylori* in mucin solutions and gels. We then discuss how the biochemical strategy of urea hydrolysis required for survival in the acid is also relevant to the mechanism that enables flagella-driven swimming across the mucus gel layer. Other aspects of the influence of *H. pylori* infection such as, altering gastric mucin expression, its rate of production and its composition, and the influence of mucin on factors controlling *H. pylori* virulence and proliferation are briefly discussed with references to relevant literature.

## Introduction

As is well known, *Helicobacter pylori* (*H. pylori*), the most abundant pathogen in the stomach causes gastritis, peptic ulcers, and gastric cancer by establishing colonies on the epithelial surface of the stomach that generate a host-immune response. It has three major pathogenic effects on its host: gastric inflammation, disruption of the gastric mucosal barrier, and alteration of gastric physiology (1–4). The virulence factors that are responsible for the pathogenic effects also enable the bacteria to manipulate the host-immune response and support its long-term survival in the stomach (5–9). The question of how the bacterium initially breaches the protective mucus barrier to reach the epithelial cell surface and colonize in the extreme acidic environment of the stomach (10) is particularly intriguing from a physical perspective. Bacterial motility in aqueous solutions is well understood (11, 12), and motility through viscous polymeric solutions has been investigated for many years (13–18). However, *much less is understood about how a bacterium moves through a gel, and how the motion depends on, and in turn, influences the structure and dynamic properties of the mucus gel*. Recently, some theoretical advances have been made on the related problem of motility of sperm [for a recent review see Ref. (19)], and on the swimming of helical objects in a viscoelastic medium (20). The presence of an elastic network with fluid filled pores in a gel raises questions such as: (i) “Can a bacterium move through a gel, and if so, how does it move?” (ii) “Is it possible for the bacterium flagella motors to exert sufficient force and torque to deform a gel and enable it to move?” (iii) How do the speed and torque depend on the rheological parameters of the gel? Conversely, does the bacterium alter the physical and chemical properties of the gel? In this review we describe the physical characteristics of the gastric mucus lining, and discuss the structure and gelation of gastric mucin, which is the gel forming component of mucus. We then address the question of the motility of *H. pylori* in the acidic environment of the stomach, and its influence on the structure and mechanical properties of the mucus gel.

The gastrointestinal (GI) tract, like other physiological systems with a cavity open to the environment, is lined with a protective mucus layer [for reviews see Ref. (21, 22)]. The high molecular weight glycoprotein, mucin, secreted by cells in the lining of these organs is responsible for giving mucus the physical characteristics of a viscoelastic fluid and a hydrogel (21). This physical state presents a unique environment to the more than a trillion bacteria that live or move in the GI tract. In the stomach, like the intestine, bacteria are found in the viscous fluid-like outer mucus layer but not in the dense mucus gel layer that adheres to the cell surface (23, 24). Compared to the intestine, relatively few bacteria inhabit the mucus layer of the stomach, as most are unable to survive in the acidic environment of the stomach (25). *H. pylori*, the most abundant, and the most long-term inhabitant of gastric mucosa, copes with the acidic environment by secreting urease to hydrolyze urea and produce ammonia to neutralize the acid (26, 27). How it gets across the mucosal barrier in the stomach to colonize on the gastric epithelium (10, 28, 29) is a largely unsolved problem. The commonly held view that it bores its way through the mucus gel-like a corkscrew (10, 30) is probably not valid, in light of observations showing that *H. pylori* are immobile in porcine gastric mucin (PGM) gels under acidic conditions, although their flagella rotate and they wiggle in-place. Instead, we show that motility across the gel is achieved due to the same biochemical mechanism that *H. pylori* uses for surviving in the acid, namely urea hydrolysis to elevate the pH of its environment. The elevation of pH to neutral transforms the viscoelastic mucin gel to a viscous liquid, enabling the bacterium to swim in the viscous solution (31). Additionally, the helical cell-shape may enable it to swim faster in the viscous solution, as implied by the theoretical prediction of (20). The helical cell-shape has also been shown to be of importance in colonization, as rod- and C-shaped mutants of *H. pylori*, although motile in soft agar, are not as effective in establishing colonies (32, 33).

*Helicobacter pylori* infection is also known to impair mucin production, and alter the composition of mucins in the gastric mucus (34). Byrd et al. (35) show that MUC 6, normally associated with gastric gland mucous cells, is expressed in surface mucous cells of *H. pylori* infected patients, while the MUC5 component of surface mucous cells decreases. Navabi et al. (36) report that MUC1 turnover and level is decreased upon *H. pylori* infection in mice. Chronic infection leads to intestinal metaplasia, with the stomach mucus developing characteristics of intestinal mucus (37). Newton et al. (38) report an 18% reduction in the amount of gel forming, high molecular weight mucin, although they noted that thickness of the mucus layer is uncompromised. On the other hand Henriksnäs et al. (39), observed a reduction in the thickness of the adherent mucus layer in mice. Mucins also bind to *H. pylori* (24, 40), the binding is pH dependent (41, 42) and the bacterium is chemotactic toward mucin. Conversely, mucins influence the proliferation, gene expression, and virulence of *H. pylori*, implying a dynamic interplay between the bacterium and its host (43). These aspects will not be further addressed in this review; the reader is referred McGuckin et al. (24) for the complex interplay between mucins and bacterial pathogens. In the remainder of this review we focus on the physical properties of mucus relevant to the transport of *H. pylori* across the mucus barrier, and provide some insight into the mechanism of *H. pylori* motility. We also address the impact of *H. pylori* infection on mucus structure and rheology.

## Structure of Mucus

Of all the organs, it is in the stomach that mucus faces its severest challenges from secreted HCl, digestive enzymes, alcohol, drugs, and bacteria such as *H. pylori* (10, 44). Gastric mucus is a highly hydrated (swollen to ∼95% water), viscoelastic substance containing 3% of mucin glycoprotein mixed with about 2% low molecular weight lipids, electrolytes, other small molecules, and other proteins such as trefoil factors (21, 45). The mucin glycoprotein is responsible for the remarkable hydration, viscoelastic, and mucoadhesive properties of the protective mucus layer (21, 46). These properties are primarily related to the ability of mucin to polymerize to high molecular weight [for reviews of biophysical properties of mucin see Ref. (47, 48)]. At the typical concentrations found in mucus secretions in mammalian stomachs, mucin further aggregates, and gels under acidic pH.

The gastric mucosal surface is coated with mucus, about 200–400 μm in thickness, consisting of an adherent layer of mucus on the epithelial surface covered with loosely attached, mobile mucus on the luminal side (49). Atuma et al. (50) reported *in vivo* measurements of the thickness of the mucus lining from stomach to colon in anesthetized rats. They observed that the mucus lining is continuous and consists of two layers, a loosely adherent outer layer which can be easily removed by suction and a firmly adherent layer attached to the epithelial surface. In the rat stomach the loose layer varies from 100 to 120 μm and the firmly adherent layer ranges from 80 to about 150 μm.

**Figure 1:**
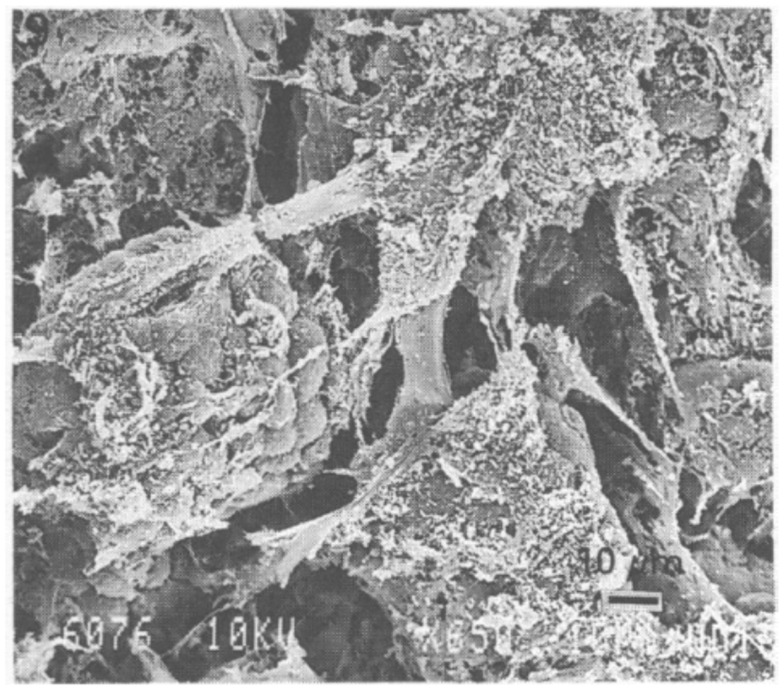
**Scanning electron microscopy image of rabbit stomach mucosa showing surface cells and numerous bundles of fiber-like mucus strands**. Scale bar 10 μm. Reproduced from Nunn et al. (51) with permission from Wiley.

Several investigators have used scanning electron microscopy (SEM) to visualize the stomach mucosal surface [see for e.g., Ref. (49, 51–53)], and numerous images and beautiful illustrations of the microstructure of the inner surface of the stomach, with and without bacteria, are readily available see for e.g., http://katierosejohnston.blogspot.com/2011/09/research-images.html. These pictures reveal a highly convoluted, self-similar, or fractal surface with numerous folds of stomach epithelia forming gastric glands (also called gastric pits) which open on the luminal surface of the stomach. Mucus secretions can be seen as wispy, fibrous material on the surface on many of these images. Forte (52) reported low-resolution images of bullfrog mucosa where the residual mucus secretion left on the SEM specimen was visible as bright white, coagulated strands. In this review, we reproduce an image from Nunn et al. (51) showing surface mucous cells, covered with sheets of mucus (Figure 1).

**Figure 2:**
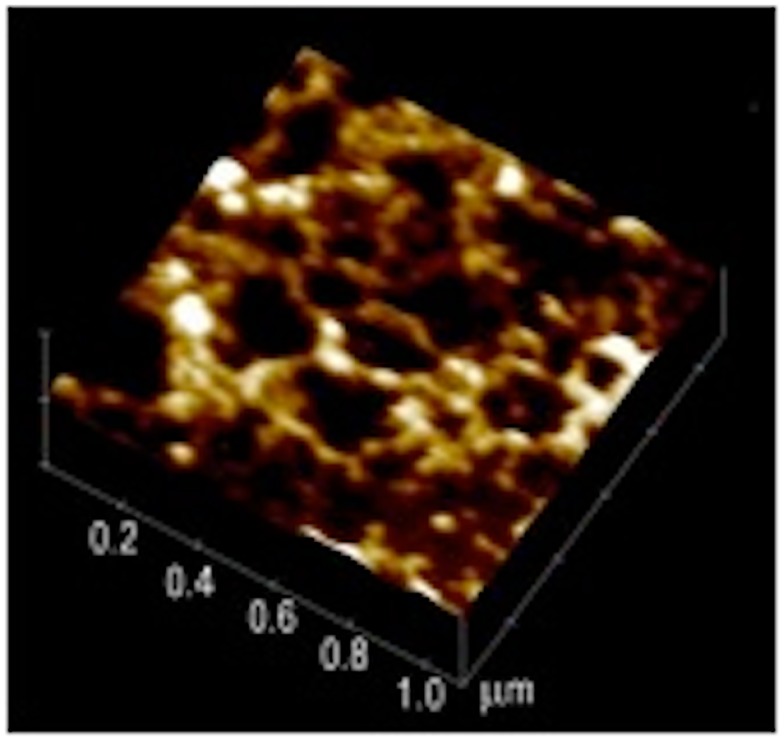
**Atomic force microscopy image of endoscopic specimen of human mucus gel**. This 1 μm× 1 μm image reveals a network with a “pearl-necklace” morphology formed by mucin aggregates. Reproduced from Hong et al. (55) with permission from American Chemical Society.

The shiny, translucent film of mucus, visible to the naked eye, can be removed by gentle scraping and further processed to prepare purified mucin (54). To avoid the perturbative effects of SEM preparation we examined the structure of hydrated mucin and mucus on the sub-micron length scale *in vitro* by atomic force microscopy (AFM) in a liquid cell under appropriate buffers. A tapping mode AFM measurement of a wet sample of human mucus taken from the discarded material obtained in the lavage following a gastric biopsy reveals a swollen network (Figure 2) formed by the glycoprotein mucin (55). The mucin appears to form aggregates that are connected as in a “pearl-necklace” and enclose aqueous pores of about 200–300 nm in diameter.

## Molecular Composition of Gastric Mucin

**Figure 3:**
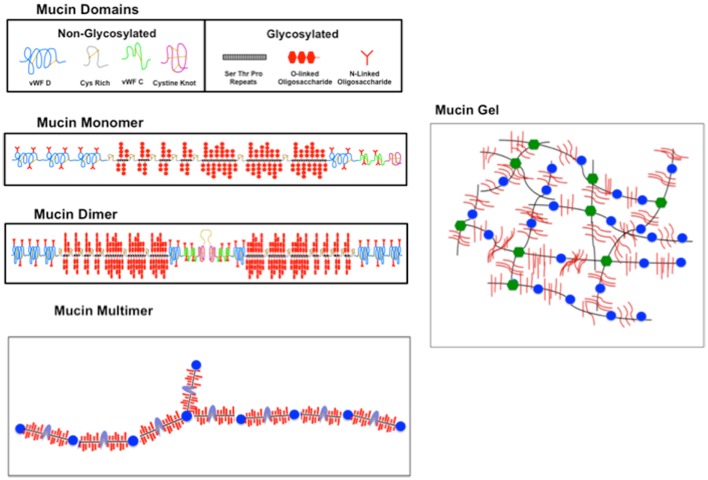
**Sketch illustrating the domain structure, polymerization, and gelation of mucin**. (Topmost panel) The constituent glycosylated (g) and non- (or weakly) *N-glycosylated* (ng) domains of mucin represented by icons as labeled. (Second panel) The g and ng domains are linked as shown to form the mucin monomer, with vWF domains at the N terminal and cysteine rich and cysteine knot domains at the C terminal, interspersed with the O-glycosylated STP repeats forming the g domain. (Third panel) A dimer formed by two monomers linked with a disulfide (S–S) bond involving the C-terminal cysteine knot domains. (Bottom panel) A multimer with alternating g (black with red brush) and ng (blue) domains. An N-terminal branch is also indicated. (Right hand panel) A sketch of a mucin gel formed by hydrophobic association of unfolded ng domains of the multimer. The crosslinking is represented by changing the color of ng domains from blue to green. New sketch based in part on Ref. (48) with permission from Elsevier.

For completeness we include a brief description of mucin composition, although this topic has been reviewed extensively. Gastric mucin, like other mucins is a very high molecular weight glycoprotein (2–20 million g/mol) with about 70–80% polysaccharides. The protein, which is quite unlike normal globular proteins, forms the linear core of the molecule on which the polysaccharide chains are radially arrayed similar to the bristles of a bottle-brush, as shown in Figure 3 [adapted from (48)].

Of the ∼20 mucin genes that have been identified with a similar sequence structure (56, 57), gastric mucus contains only two secretory mucins MC5AC and MUC6, in addition to the ubiquitous cell surface mucin MUC1 (58). Dekker et al. (59) suggest that the mucin family be separated into two groups on the basis of sequence homology, one group representing those similar to the cell surface mucin MUC 1, and the other group comprising the secretory mucins similar to MUC2. Both MUC1 and MUC2 mucins contain large domains comprised of tandem repeating sequences of serine (S), threonine (T), and proline (P), located in the heavily *O*-glycosylated portion of the molecule. The S and T amino acids provide the O-glycosylation sites for the covalent attachment of the polysaccharide. The length and number of the STP repeat varies for the different mucins coded by different genes, and also varies between species. For example, MUC5AC contains 66–124 repeats of 8 amino acids with the consensus sequence TTSTTSAP (60) and MUC6 contains 15–30 repeats of 169 amino acids (61). In all MUC2 type secretory mucins, this glycosylated (g) domain occupies the central region of the apoprotein. It is flanked by cysteine rich domains and a Cystine Knot domain, with non-repeating sequences at the C-terminal (see Figure 3), and by domains similar to the von Willebrand Factor (vWF) C, D domains involved in blood clotting pathways, at the N-terminal*.* These weakly or non-glycosylated (ng) regions resemble typical secreted globular proteins in their amino acid composition and contain small amount of isolated *N*-glycosylated oligosaccharides, but do not form a bottle-brush.

## Polymerization, Aggregation, and Gelation of Mucin

Early studies of mucin were interpreted in terms of tetrameric wind-mill like structure (45). However, transmission electron microscopy (TEM) studies clearly established that gastric, cervical, and respiratory mucins are all linear polymers (62, 63). AFM and dynamic light scattering (DLS) studies further show that even in solution, mucins are highly elongated, rod-like, or worm-like polymers [for detailed review and references see Ref. (47, 48)]. AFM imaging of individual molecules of PGM in aqueous solution reveals long, curvilinear filaments ranging from 500 nm to 4 μm in length (55) and ∼1 nm in height, reflecting the height of the hydrated brush probably flattened down due to interactions with the AFM tip. The height and diameter reported by Hong et al. (55) were made in an aqueous environment, and thus provide a better estimate of the dimensions of the hydrated, native PGM molecule than that obtained by TEM measurements (63) or earlier AFM measurements (64) which were done on dried films. Hong et al. (55) also examined the pH dependence of PGM, observing *in situ* aggregation at pH < 4 with the formation of large, spherical, or oblong aggregates and a 5- to 10-fold increase in height.

The formation of large glycoproteins with molecular weight ranging from 2 to 20 million g/mol is generally believed to involve C-terminal dimer formation of the apoprotein *via* disulfide (S-S) linkages of the Cysteine knot domains (57, 65). These dimers then further polymerize to form large multimers, as illustrated in Figure 3. There is also the possibility of the C-linked dimers to form trimers *via* N-terminal S-S linkages involving the vWF D domains. This has been observed in MUC2 (66, 67) and in porcine submaxillary mucin (57) predominantly composed of MUC5B. This would imply the occurrence of tri-functional branches in mucin. It is not clear whether vWF D linked trimers are formed in MUC5AC, although other cleavage sites at the C-terminal have been noted (68, 69). Since TEM and AFM images show predominantly linear polymers, it is possible that trimers, if there, are present in small quantities, and the weak branching at the molecular level is not resolvable by TEM and AFM techniques.

The molecular mechanism of crosslinking in PGM gels is not fully understood. Using fluorescent dye binding (70) suggested that aggregation/gelation at low pH involves a complex interplay between electrostatic and hydrophobic interactions (48) with the formation of non-covalent crosslinks *via* the hydrophobic association of specific regions of *ng* domains, modulated by pH dependent changes in the electrostatic interactions of charged amino acids in the N- and C-terminal regions of the apoprotein (48). This network is schematically illustrated in Figure 3, which shows the mucin polymer as having long glycosylated (g), hydrophilic domains, alternating with short, and somewhat hydrophobic ng domains. The differential affinity for water of the alternating domains will stabilize non-covalent crosslinks formed by association of hydrophobic amino acids exposed at low pH in the ng domains in the C- and N-terminal regions, as illustrated in Figure 3. Exactly which domains are involved in these hydrophobic interactions, and whether it involves single domains from dimers or whether it involves the N-terminal tri-functional vWF units has not been explored. Discrete molecular dynamics simulations show pH induced changes in the folding of the PGM 2X domains that are not seen in vWF C domains (71). Further work, both theoretical and experimental, concerning the folding and association of the ng domains, and the swelling of the gel due to the electrostatic interactions of the negatively charged polysaccharide brush of the glycosylated domains, would be valuable in developing a detailed molecular model of pH induced gelation of mucin.

## pH Dependent Viscoelastic Behavior of Mucin

**Figure 4:**
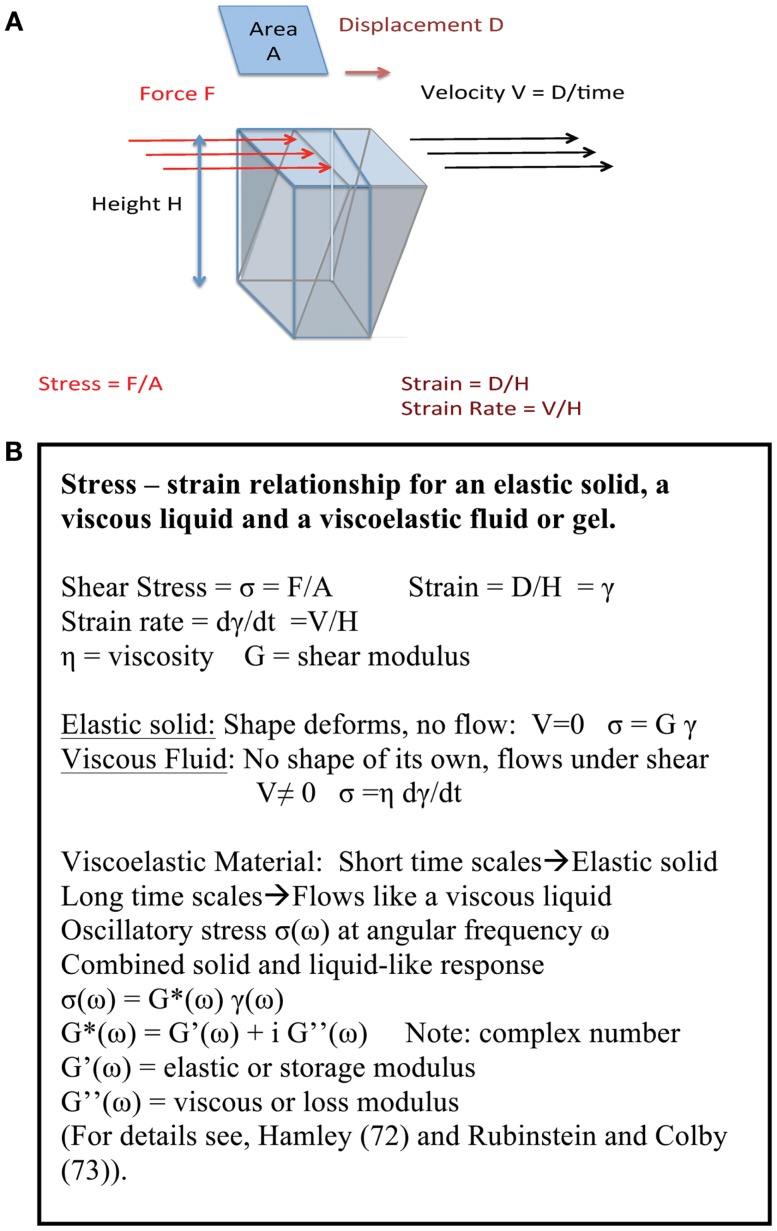
**(A) Key concepts of viscoelasticity**. A rectangular object with a surface area *A* and height *H*, fixed at the bottom surface, is subjected to a shear force *F* by pushing along the top surface. If the object is a regular solid then the top surface displaces by an amount *D* deforming the object. If the object is a liquid between two plates, with the bottom plate fixed and the top plate pulled at a speed *V*, it flows with speed increasing from 0 at the bottom to *V* at the top, so the deformation is given by *V*/*H*. If it is viscoelastic, both solid-like and liquid-like deformations occur simultaneously, *albeit* on different time scales. The origin of viscoelasticity lies in the ease with which polymer chains can be deformed, and the fact that their motion is controlled by transient entanglements and long-lived crosslinks between chains. **(B) Summary of stress – strain relationship**. The basic definitions for an elastic solid, a viscous liquid and a viscoelastic fluid or gel are summarized here.

The formation of a network in gastric mucin at low pH has a profound influence on its rheological properties, i.e., its fluid-like flow behavior and response to mechanical deformation and shear forces. In view of the key role that these properties play in the transport of particles and bacteria across mucus, we discuss the underlying concepts before discussing the results. Like many other soft biological materials, the mucus layer exhibits a viscoelastic response to deformation that represents the combined effect of both its liquid-like and its solid-like features, arising from the presence of a polymer network filled with liquid. As illustrated in Figure 4A, a normal viscous solution, like water or glycerin, flows when subjected to a tangential shear force with a viscosity η, reflecting the resistance to flow, i.e., the dissipation or loss of energy. In contrast, an elastic solid stretches (or compresses) like a spring when subjected to tensile forces, and its shape deforms, like the spine of a hardcover book, when subjected to shear forces, but it does not flow in either case. A viscoelastic material exhibits both liquid-like flow and solid-like elasticity, as summarized in Figure 4B (72, 73). One of the hallmarks of a viscoelastic material is that its response to deformation depends on the time for which the material is deformed, as illustrated by the familiar Silly Putty which bounces like a ball when dropped quickly (reflecting the solid-like response on short time scales), but flows like a liquid when stretched slowly (reflecting the large time scale liquid-like flow). The text box lists the basic relations of viscoelasticity.

A very simple assay of pH dependence of viscosity of mucin was reported by Bhaskar et al. (54) by measuring the terminal speed^1^ of a micron sized steel ball falling under gravity in purified PGM mucin solutions. The ball falls slower as the pH is reduced, and does not fall at all in mucin at pH 2, indicting the formation of a gel network at pH 2 (54).

**Figure 5:**
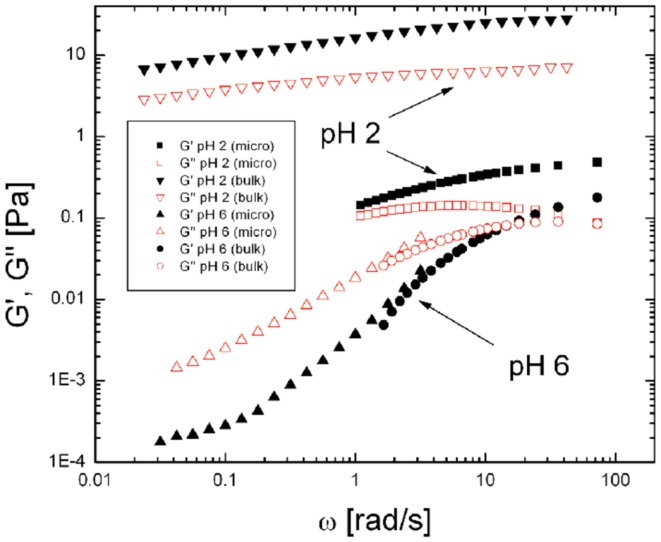
**Comparison of viscoelastic moduli, *G*′(ω) and *G*′′(ω) for PGM at pH 2 and pH 6 probed by bulk rheology and particle tracking microrheology**. In the solution state (pH 6 PGM) bulk and microrheology measurements of both components, *G*′(ω) and *G*′′(ω) (labeled as indicated in the Figure) are in agreement over the frequency range accessible. In contrast, the viscoelastic moduli obtained by bulk and microrheology differ significantly in the gel phase (pH 2) suggesting the presence of microstructural heterogeneity and length-scale dependent rheology in the gel state. Reproduced from Celli (80) with permission of author.

Further insight into the rheological properties of a gel can be obtained from techniques such as oscillatory shear rheology which provides a direct measurement of the frequency dependent bulk viscoelastic moduli of a material. Oscillatory shear measurements clearly show that viscoelastic properties of gastric mucin are highly pH dependent, as shown in Figure 5 (74). At elevated pH (>4) gastric mucin flows like a viscous polymer solution, in which mucin glycoprotein macromolecules are in solution phase, with only transient intermolecular entanglement. In these conditions response to deformation is dominated by viscous flow (and viscous modulus *G*′′(ω) > elastic modulus *G*′(ω), over a wide range of values of ω) with a small (but measurable) elasticity. Conversely, at pH < 4, associations between mucin domains give rise to a connected intermolecular network with significant elasticity and minimal flow [elastic modulus *G*′(ω) > viscous modulus *G*′′(ω)] (see text box for definitions). Further insight is gained by examining the scaling relationship in plots of *G*′(ω) and *G*′′(ω) versus ω and identifying the frequency at which the moduli cross over as described previously (74, 75). This behavior is paralleled in a wide range of biopolymer systems driven by polymer concentration, solvent interactions, pH, salt concentration. Similar results on the pH dependence of PGM have been reported by Maleki et al. (76) using rheo-SALS, a rheometric technique coupled to small angle light scattering.

Bulk rheology, as described above, provides the average viscoelastic response of a material. However, in a swollen gel such as mucin, the elastic, and viscous response is likely to be length scale dependent due to the inherently heterogeneous structure of a gel consisting of fluid filled pores and network strands. The local, microrheological properties can be probed by tracking the hindered, Brownian motion of micron sized polystyrene latex particles in the medium of interest and calculating the complex elastic modulus G*(ω) from the mean square displacement ⟨Δr^2^⟩of the particle (77–79). Using this method, Celli (80) found that both *G*′(ω) and *G*′′(ω), the elastic and viscous moduli, at pH 6 have similar magnitudes to those obtained in bulk rheology, as expected for particles moving in a viscous solution (80). In contrast, at pH 2 the moduli obtained by microrheology were significantly lower than those obtained by bulk rheology (Figure 5), indicating that, on small length scales, the particles sample a less viscous environment than the bulk gel. Similar results were obtained in a microscopic DLS study (81) in which the much smaller, 100 nm latex particles, appeared to probe two microenvironments; with some beads moving freely in large pores in the pH 2 gel, and others displaying the same slow relaxation as the signal from the dynamics of the gel itself. Lieleg et al. (82) also observed heterogeneities in their investigation of particle translocation through mucin hydrogels.

**Figure 6:**
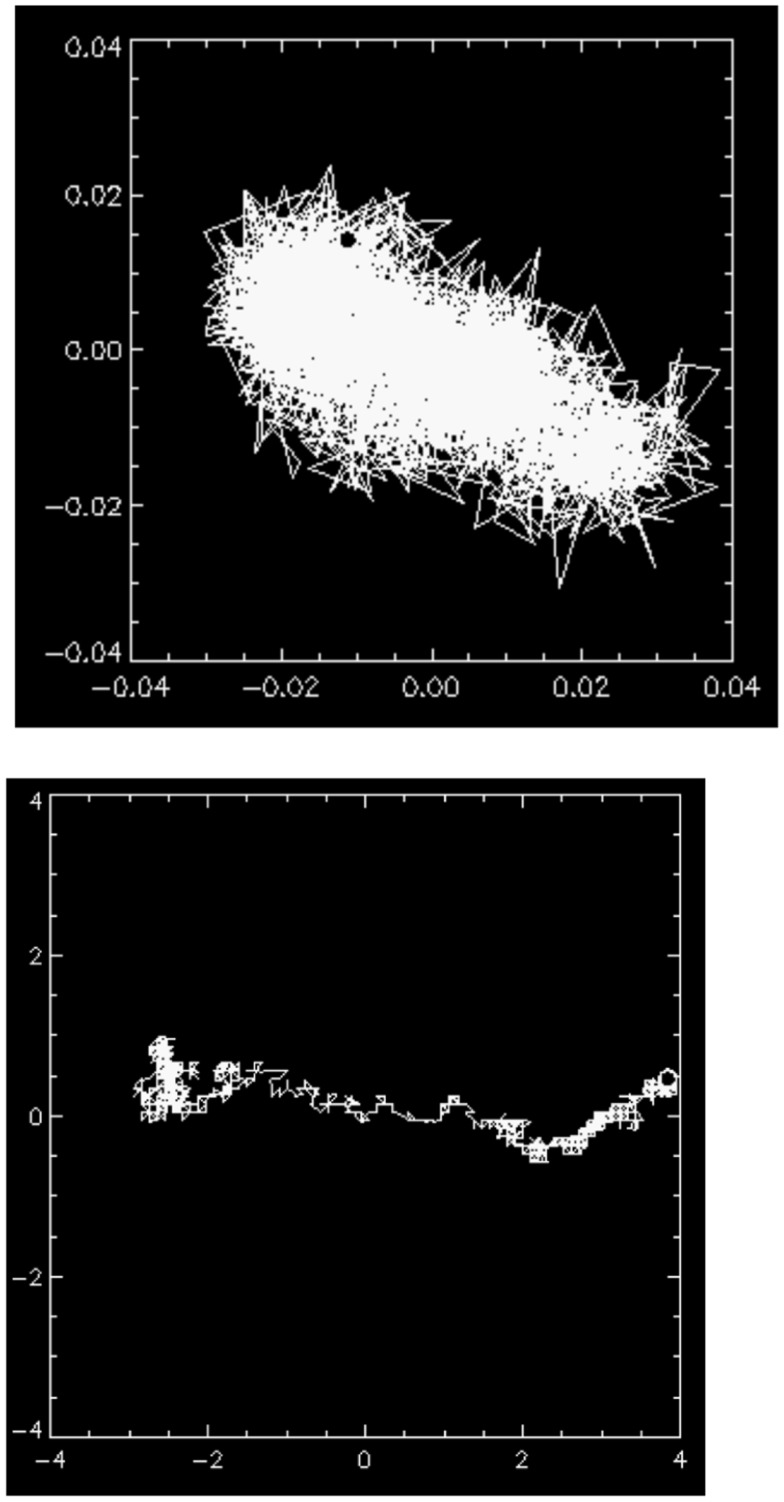
**(Top) the motion of the center of mass (c.m) of a 1 μm latex bead trapped in a pore in the mucin gel at pH 2**. The maximal excursion of the particle’s c.m <0.1 μm, indicating that it is essentially immobile in the gel network. (Bottom) occasionally, a particle finds a channel in the gel network indicating that the network structure is heterogeneous. The length unit in both images is micrometer. Reproduced from Celli (80) with author’s permission.

Time-resolved microscopic particle tracking measurements (80) show that at pH 2 the probe particles move in an inhomogeneous micro-environment consisting of water filled pores in a gel and thus encounter reduced viscous damping. This is illustrated in Figure 6 showing the trajectory of the center of mass (c.m) of a 1 μm latex bead trapped in a pore in the pH 2 mucin network, and only moving at most a total distance of ∼0.1 μm. Rheological studies under non-linear deformations reveal an apparent yield stress, the stress at which the elasticity breaks down, that is also highly influenced by pH. The gel begins to flow just above 10 Pa (74). PGM exhibits a highly non-Newtonian shear thinning behavior, viscosity decreasing with increasing stress in steady shear flow tests, as was previously observed in commercially made PGM (83). The lower pH samples are dramatically shear thinning, decreasing in viscosity by about three orders of magnitude over four decades increase in shear rate and approaching a constant yield stress at low shear rates. These findings on purified mucin are consistent with bulk and microrheological measurements of mucus rheology (84), and provide a molecular basis for understanding the rheological properties of mucus. The shear thinning and yield stress may be relevant to mucus shedding during peristalsis, and could provide a physical mechanism for washing away bacteria that are not adhered to the epithelial surface. It would be interesting to investigate the rheological properties of the mucin from the two distinct mucus layers discussed earlier in this review (50).

We end this section by a cautionary remark concerning the use of commercial mucin preparations such as those obtained by Sigma Aldrich. These do not form a pH dependent gel, because the mucin has been proteolytically digested during purification (85). Such a reduced mucin does not gel upon lowering pH (54). The rheological behavior also depends on which mucin is the predominant component of the preparation. For example, the Orthana MUC6 mucin investigated by Yakubov et al. (86) and Di Cola et al. (87) has a linear dumbbell structure with a central glycosylated portion flanked by hydrophobic, ng regions, suggesting that it consists of monomeric apoprotein, and not the polymerized, gel forming mucin (c.f. Figure 3).

## Motility of *H. pylori*

The work described above on particle diffusion in gels shows that the microstructure of mucin and mucus gels restricts the diffusional motion of micron sized particles in such gels. It is not *a priori* clear how the flagella-driven motion of *H. pylori* would be impacted by the size restriction due to the movement in a confined geometry. In the next part of this review we address how the helical shape of *H. pylori* and it’s unique biochemical adaptation to survive in an acidic environment also enables it to go across the mucus gel.

Like most other bacteria that colonize the GI mucosa, *H. pylori*, too have evolved to be adapted to their unique niche of the stomach mucosa. These Gram-negative, helical-shaped bacteria, 2.5–5.0 μm in length and 0.5–1.0 μm wide (88) have four to six unipolar-sheathed flagella, which are essential for bacterial motility. Each flagellum, ∼3 μm long and 2.5 nm thick, has a characteristic terminal bulb, which is an extension of the flagellar sheath (88). The motility and the helical shape have both been shown to be of importance to the survival of these organisms. Eaton et al. (89) showed that flagellar mutants of *H. pylori* were unable to colonize the gastric mucosa of gnotobiotic piglets. More recent work by Ottemann and Lowenthal (90) establishes that mutants with non-motile but otherwise intact flagella also do not colonize.

*Helicobacter pylori* has also evolved to survive in the acidic environment of the stomach (91). It is well established that the ability of *H. pylori* to hydrolyze urea and elevate the pH of its surroundings is important in enabling it to escape the acidity of the gastric lumen (26, 27, 92, 93), penetrate the thick mucosal gel and reach the surface epithelium (91). *H. pylori*, exhibits chemotaxis toward urea present in the epithelial cell surface and a pH tactic response toward elevated pH, both of which may also be crucial for survival in the stomach (94). *H. pylori* survival in acidic conditions is also stated conversely, namely that acidic conditions are required for *H. pylori* survival in the presence of urea because the subsequent rise in pH to highly alkaline levels is also toxic to the bacterium. To help avoid overproduction of ammonia, the urea channel is regulated by protons to open at low pH and close at high pH. *H. pylori’s* TlpB receptors enable pH taxis (95, 96). It uses the mucosal pH gradient which varies from low on the luminal surface to neutral on the cell surface (28, 97) to move away from the lumen toward the mucosal surface (98) where it attaches itself with adhesins (10, 99). Mutants lacking either the TlpA or TlpB receptors also show altered extent of inflammation (100). The growth of *H. pylori* in culture is also pH dependent (101).

The motility and chemotaxis of *H. pylori* and the related *Campylobacter* has been investigated by using microscopic tracking [for a recent review see Ref. (102)]. By detailed comparison of the motions of straight-rod *E. coli* and helical bacteria *H. pylori* and *Campylobacter*, in liquid cultures Karim et al. (103) showed that the helical bacteria swim faster than *E. coli*, presumably due to their helical body shape. Their finding is consistent with the idea of Berg and Turner (16) that a helical shape would result in additional screw-like propulsion for bacteria moving in viscous environments such as those faced by *H. pylori* in its native environment. However, differences between species cannot be ruled out in this comparison. Yoshiyama et al. (30, 104) and Worku et al. (105) investigated the motility and chemotactic response of *H. pylori* in viscous synthetic polymer solutions, and found that swimming speeds *decrease* with *increasing* viscosity of the polymer solution, and the bacteria became immobile at very high viscosities.

## Motility of *H. pylori* in Mucin Solutions and Gels

**Figure 7:**
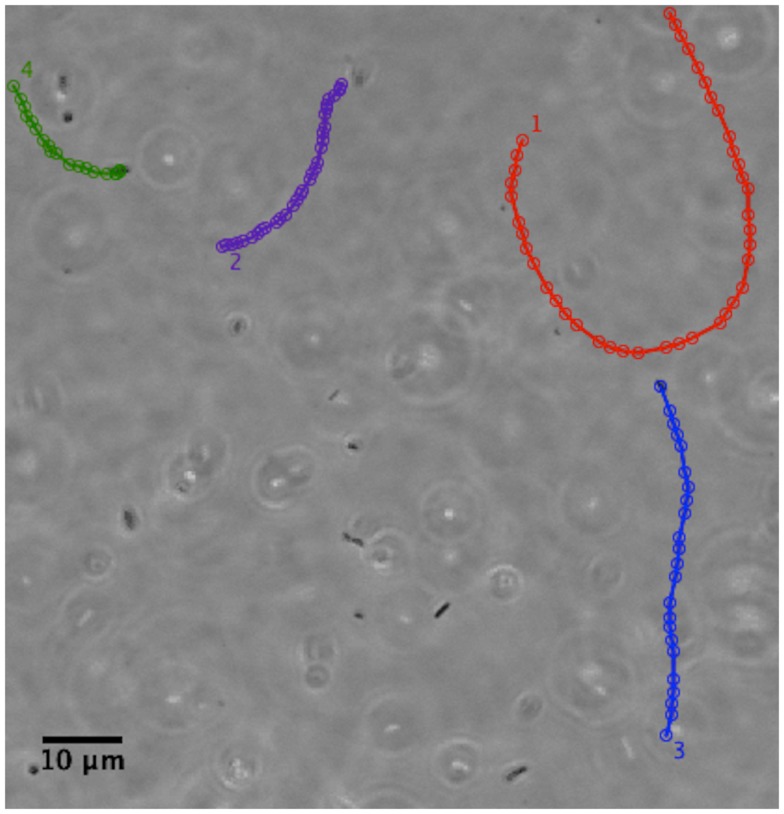
**Phase contrast image showing the tracks of a few *H. pylori* bacteria in a PGM solution 15 mg/ml at pH 6**. Adapted from the movies included as supplementary material in Celli et al. (31). The tracks have been colored for ease of visualization. Permission not required for author’s re-use of their own work in PNAS after January 2009. Similar tracks are also shown in Celli (80).

We have examined the motility of *H. pylori* in purified gastric mucin at different pH’s mucin (31) using phase contrast, digital video microscopy to image live bacteria. Some typical results from analysis of the movies published by Celli et al. (31) are shown and discussed here. We observed that in PGM at neutral pH of 6 or 7 the bacteria swam considerable distances, along almost linear or curved tracks as shown in Figure 7. They exhibit a large spread in their speeds, reflecting both the variation in the size/shape and number of flagella of individual bacteria, as well as the variation in their speed as flagella motors fire asynchronously, and flagella bundle and unbundle. The mean speed averaged over all bacteria is about 16 μm/s which is qualitatively comparable to the speeds of about 20–30 μm/s reported by Worku et al. (105) in methylcellulose.

In contrast to the swimming behavior observed in PGM solution at pH 6–7, we noted that when bacteria were added to PGM gels buffered at low pH of 4 or 2 and deprived of urea, they were immobilized and did not move over any measurable distance. Rotation of the flagella and wiggling of the bacteria in-place could be observed at 60–100× magnification, but this did not displace the c.m of the bacterium. Using theoretical models based on resistive force theory of Magariyama et al. (106–108) we obtained the motor torque as 3.6 × 10^−18^ Nm (31), which is about three times the torque of *E. coli* swimming in an aqueous environment. The higher torque maybe needed to rotate the flagella in a medium.

We also noticed that, although low pH impairs the flagella motors (109, 110), the bacteria were alive at low pH over the duration of the experiment, as confirmed by increasing the pH back to neutral (see below) and observing the renewed motion of bacteria that were stuck in the low pH environment. In our experiments with motility in PGM gels, we observed a dramatic difference depending on the presence of urea. In the absence of urea the bacteria remained immobile in the low pH gel. However, if urea was present and the sample was not buffered, then bacteria that were immobile in a low pH PGM gel, became mobile within a few minutes. Using two-photon fluorescence microscopy to image the bacterial in an initially low pH mucin gel containing urea we showed that the onset of bacterial movement is directly correlated with a rise in pH to near neutral values, as indicated by the pH sensitive fluorescent dye, BCECF (31). These observations in purified PGM gels are consistent with observations in anesthesized gerbils, which showed that *H. pylori* became immotile in <1 min at lumen pH values of 2 and 3, and in 2 min at pH 4, but remained motile for more than 15 min at pH 6 (111).

## Infection with *H. pylori* Affects the Rheological Properties of Purified PGM

**Figure 8:**
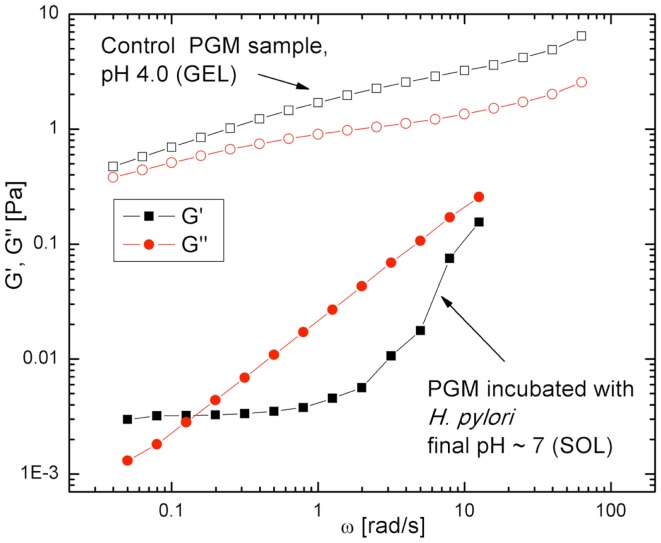
**Frequency dependent viscoelastic moduli, for *H. pylori* infected PGM and control sample, both at pH 4 initially**. The elastic response *G*′ (open black symbols) and viscous response *G*′′ (filled red symbols) data show that the control sample is a gel (*G*′ > *G*′′) while the infected sample is a solution (*G*′′ > *G*′). From Celli et al. (31). Copyright permission not required per PNAS policy effective January 2009.

**Figure 9:**
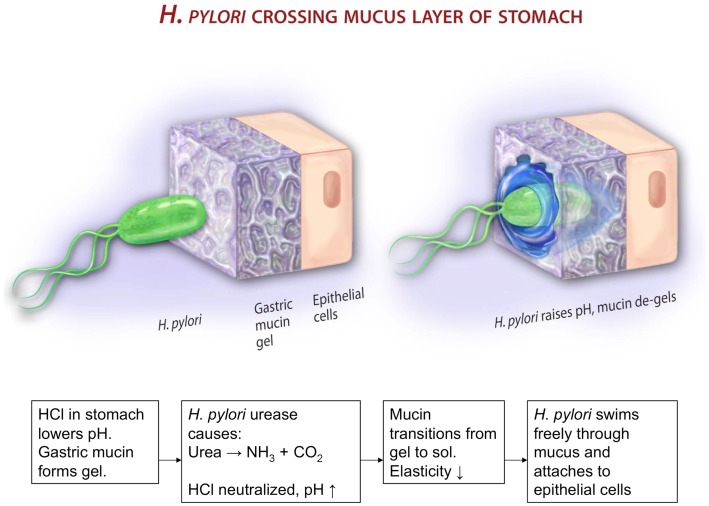
**This cartoon illustrates a possible mechanism by which *H. pylori* gets across the mucus gel**. According to Celli et al. (31), gastric mucin forms a gel at low pH < 4. The bacterium cannot move in mucin gels. It secretes urease that metabolizes urea, producing ammonia and elevating the pH. This de-gels the mucin, and enables the bacterium to swim in the resulting polymer solution. The picture, not copyrighted, is reproduced from an NSF press release 09-149. http://www.nsf.gov/news/news_summ.jsp?cntn_id=115409&org=NSF&from=news

The results above show that the motility of *H. pylori* depends on the physical state of the mucin gel. To address the converse question, whether the infection by *H. pylori* impacts the physical properties of mucin or mucus, we performed measurements of the frequency dependent bulk viscoelasticity of purified PGM infected with the bacteria using oscillatory shear methods (31). In earlier studies with purified PGM (shown in Figure 5) we had shown that at pH 4 and 2 purified PGM is a gel with an elastic modulus that dominates over the viscous response, while at pH 6 it is a solution (74). A similar experiment on a 15 mg/ml PGM sample initially at pH 4 incubated with *H. pylori* for 24 h (Figure 8) clearly shows that the rheological parameters of the infected sample were close to those of pure PGM at pH 6–7 and it remained a solution even at the lowest stress values with a greatly reduced viscosity (31). The concurrent gel to solution transition with the onset of mobility in PGM containing urea suggests that *H. pylori* gets across the mucus gel using urease secretion to neutralize the acid, raising the pH, and triggering a gel-sol transition. This is illustrated in Figure 9.

Some cautionary remarks about motility and rheology measurements are in order here. Motility measurements in a population of bacteria are inherently polydisperse. Thus, characterizing the behavior by a single average velocity provides limited information and could lead to erroneous predictions; either several different statistical measures such as median, mean, maximal, and minimal speeds should be reported or better yet, the entire distribution. It is also important to analyze hundreds or thousands of tracks, as bacteria move out of the focal plane, and sometimes get stuck in air bubbles and similar defects. By the same token, measurements made very close to the surface of the slide are likely to be influenced by substrate interactions. We also note that bulk rheology provides the average dynamical response, which may not be the same as observed in microrheology, particularly in microstructured environments. The bacteria can also find channels and swim through other heterogeneities in the medium. Bulk rheology experiments require a large sample volume (1–5 ml), while microrheology done on a microscope slide requires only a few microliters. This is a great advantage when analyzing purified samples, mutants, or expressed proteins that are available only in small quantities.

The results discussed above suggest that other factors that can de-gel the mucin would also enable *H. pylori* to get across the mucus barrier. Worku et al. (105), had observed that *H. pylori* which were immobile in biopsy samples of mucus, became motile when saline was added to the mucus gel. On the basis of our experiments showing that mucin does not gel at low pH in high salt concentrations (54, 70, 74) we suggest that saline restores motility because it too triggers a gel to solution transition. Whether these observations have implications for identifying factors such as high salt diet, that may promote *H. pylori* infection in certain populations, remains to be seen.

## Summary and Future Outlook

The review presented here focuses narrowly on the interplay between mucus structure and rheology and the motility of *H. pylori*. Among the many topics that we have not discussed are motility in a chemotactic environment, possible chemical interactions of the bacterium with mucin or other factors present in mucus, the binding of mucin to *H. pylori*, the role of *H. pylori* in altering mucin production or proteolytic digestion of mucin, the effect of the bacterium on mucous producing cells, the mucosal factors that are involved in the adhesion of the bacterium to the epithelial surface, and cell signaling in the mucus environment to form a colony. Some references on these topics have been provided.

The work discussed here however, does show the importance of physical limitations of the mucus gel microstructure and its pH dependence on the motility of this important pathogen. It also shows the usefulness of microscopic bacterial and particle tracking tools for examining the initial stage of bacterial penetration. We hope that this review encourages further theoretical investigations of the fundamental problem of addressing how a helical-shaped bacterium with helical flagella might propel itself in a viscoelastic medium. Further experimental investigations using microfluidics for investigating stomach mucus barrier (112) and single molecule imaging methods, combined with advances in molecular biology, genetics, immunology, and the availability of mutants with specific molecular and functional alterations, will enable deeper understanding of this fascinating problem.

## Conflict of Interest Statement

The authors declare that the research was conducted in the absence of any commercial or financial relationships that could be construed as a potential conflict of interest.

## Footnotes

^1^Terminal speed denotes the final speed attained after the initial gravitational acceleration is damped out by viscous forces.
